# Comparative Metabolomic Analysis of Moromi Fermented Using Different *Aspergillus oryzae* Strains

**DOI:** 10.3390/molecules27196182

**Published:** 2022-09-21

**Authors:** Seung Wha Jo, Ji-Hyun An, Dong-Shin Kim, Eun Jung Yim, Hyeon-Jin Kang, Hyun-Jin Kim

**Affiliations:** 1 Microbial Institute for Fermentation Industry, Sunchang 56048, Korea; 2 Division of Applied Life Science (BK21 Four), Gyeongsang National University, Jinju 52828, Korea; 3 Institute of Animal Medicine, Gyeongsang National University, Jinju 52828, Korea; 4 Department of Food Science & Technology, Gyeongsang National University, Jinju 52828, Korea

**Keywords:** *Aspergillus oryzae*, metabolomics, moromi, quality characteristics, sensory evaluation

## Abstract

*Aspergillus oryzae* (*A. oryzae*) is an important starter in the fermentation of koji and moromi. However, the effect of different *A. oryzae* strains on the quality of moromi has rarely been studied. For this reason, this study analyzed the physicochemical properties, enzyme activity, sensory quality, and metabolite profiles of moromi samples fermented using two strains (*A. oryzae* KCCM12012P (moromi-1) and KCCM12804P (moromi-2)), which were newly isolated from fermented soy foods, and compared them to those of a commercialized *A. oryzae* strain (control). Amino-type nitrogen contents of moromi-1 and moromi-2 samples were higher than that of control moromi, and their amylase and protease activities were also higher. Moreover, metabolite profiles of moromi were significantly altered according to strains. In particular, the levels of many amino acids, peptides, nucleotides, and acidic compounds were altered, which resulted in changes in the sensory quality of moromi. Although volatile compounds were not investigated, the results suggested that the quality of moromi was significantly different for newly isolated strains, especially *A. oryzae* KCCM12804P, and they were superior to the commercial strain in terms of taste-related substances. Therefore, these strains could be used as good starters to produce moromi and soy sauce with good sensory quality.

## 1. Introduction

Fermented foods, which have become an important part of the diet globally, are produced by means of various controlled microorganisms. During fermentation, large molecules, including proteins, carbohydrates, and lipids in the fermenting materials, are generally broken down into smaller molecules by enzymes released from microorganisms, while secondary plant metabolites can be converted to other compounds [1,2]. The fermentation process gives fermented foods a unique flavor, enhances nutritional value, and improves functional properties [3,4]. Moreover, fermented food intervention studies and large cohort studies have found that the consumption of fermented foods is positively associated with a reduction in the risk of obesity-related diseases, cardiovascular diseases, diabetes, and overall mortality [5,6]. There are many different types of fermented foods according to fermentation methods and materials, such as dairy products, meat, fish, and plants, such as vegetables, fruits, and beans [7,8].

Among fermented foods, soy sauce, with its strong umami taste, is one of the most important liquid condiments, and it has been traditionally made from fermented soybean paste, grains, and brine using mold, such as *Aspergillus oryzae* (*A. oryzae*) and *Aspergillus sojae* [9], although some commercial soy sauces are made by fermentation or by chemical hydrolysis [10]. The process of making soy sauce varies from one country to another, but in general, it undergoes the following three stages: koji, moromi fermentation, and aging [11].

Koji is produced by mold inoculation, such as *A. oryzae* and *Aspergillus sojae*, on roasted soybean and wheat [11]. Koji is continuously soaked in brine and fermented for several months to 4 years, and this process is called moromi fermentation [11]. The fermented moromi is aged for months to years to produce a unique taste and soy sauce flavor [12]. In these processes, koji and moromi fermentation are important steps because the physicochemical and flavor properties of moromi fermented by mold are closely correlated with the quality of soy sauce [11]. Moromi fermentation is generally affected by many factors, including fermentation conditions, raw materials, and fermentation period, but mold is the most significant one [10,11]. Furthermore, among various molds, *A. oryzae* plays an important role in the production of koji and moromi fermentation [11,13].

*A. oryzae*, a safe filamentous fungus recognized by the Food and Drug Administration and the World Health Organization [14], is widely used as a starter for koji production [14] because it secretes enzymes needed to hydrolyze soy polysaccharides and proteins without the production of aflatoxins [15,16]. Various *A. oryzae* strains have been isolated and selected for soy fermentation, and their general characteristics and effect on the quality of fermented products have been widely investigated [17,18]. Previous studies examined the effect of different *A. oryzae* strains on the quality of fermented foods, such as rice wine [19], soy sauce [20], and soybean koji [21]. However, the effect of *A. oryzae* strains on the production of sensory-related metabolites and taste quality of moromi has rarely been studied. 

Therefore, in this study, two new *A. oryzae* strains were isolated from fermented soybean paste, and the quality of moromi fermented by each strain was compared with that of a commercial strain (*A. oryzae* KACC47838) [22]. The general characteristics, sensory properties, enzymatic activities, and metabolite profiles of moromi fermented by different *A. oryzae* strains were analyzed, and their correlation was investigated. Moreover, the metabolomic pathway of moromi with different *A. oryzae* strains was proposed.

## 2. Results and Discussion

### 2.1. Isolation and Identification of A. oryzae

**Figure 1:**
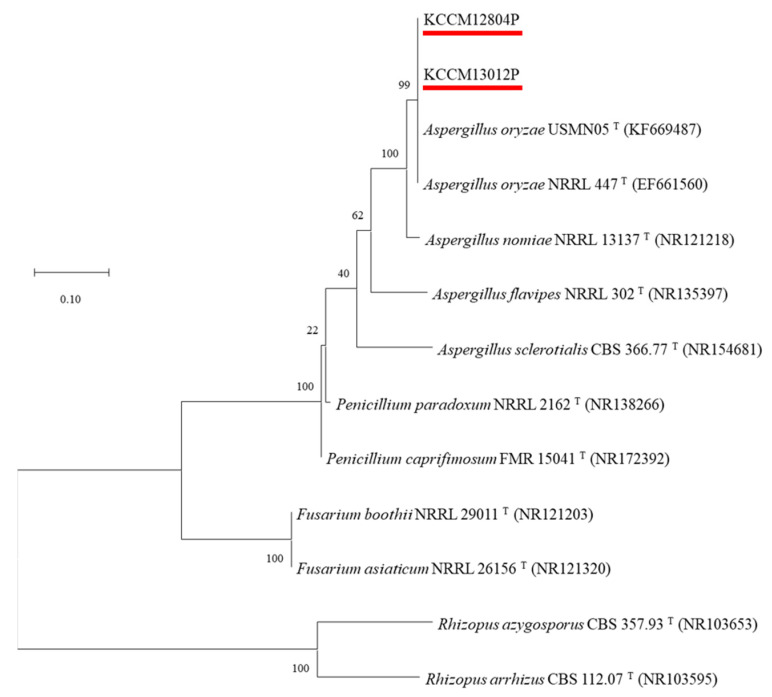
Phylogenetic tree based on internal transcribed spacer sequence (ITS-1) of KCCM13012P and KCCM12804P, and other *Aspergillus* type strains. GenBank accession numbers are given in parentheses and the branching pattern was generated by the maximum likelihood method. Bar means 0.10 substitutions per nucleotide position, and bootstrap values are expressed as percentages of 1000 replicates.

Two mold strains isolated from traditional soybean paste were identified by ITS gene sequencing (Figure S1). Gene sequencing of two isolated molds showed 99% similarity with that of *A. oryzae* strains, including USMN05T (KF669487), NRRL447T (EF661560), and NRRL13137T (NR121218). In addition, two isolated molds belonged to *A. oryzae* by the phylogenetic tree constructed with the ITS gene sequencing, and they were named *A. oryzae* KCCM13012P and *A. oryzae* KCCM12804P (Figure 1). *Aspergillus* species are mostly founded in fermented soybean products, including soy sauce [23] and soybean paste [24], and they are recommended as an ideal mold for the fermentation industry because of their various secretory hydrolytic enzymes and non-pathogenic properties [16].

### 2.2. Physiochemical Properties and Enzymatic Activities

The physiochemical properties of moromi fermented with different *A. oryzae* strains are presented in Table 1. The pH of the moromi samples fermented with *A. oryzae* KCCM13012P (moromi-1) or KCCM12804P (morimi-2) was 6.44 and 6.17, respectively, and their total acidities were 2.39% and 2.41%, respectively. These values are not much different from those of the control moromi (pH 6.41 and 2.08%). However, the amino-type nitrogen in moromi-1 (2061.93 mg%) and moromi-2 (2221.47 mg%) was higher than that of control moromi (1175.11 mg%), whereas the salinity and moisture content of samples did not indicate a significant difference. The color of moromi was significantly different according to *A. oryzae* strains. Compared with L*, a*, and b* values (82.07, 2.16, and 13.48, respectively) of the control moromi, moromi-1 was brighter in color, and its color values were 89.00 (L*), 0.29 (a*), and 11.75 (b*), whereas moromi-2 with 68.38 of L*, 6.89 of a*, and 19.19 of b* was darker in color. In addition, the browning index of moromi-2 (39.81) was 2.0 and 2.8 times higher than that of control moromi (19.50) and moromi-1 (14.07), respectively. In general, the browning during soybean fermentation, including moromi, is related to non-enzymatic browning, the Maillard reaction, which occurs from the reaction between hydrolyzed amino acids and sugars from raw material by microbial enzymes [25]. In particular, the degree of browning in fermented food depends on the abundance of amino acids and sugars hydrolyzed by enzymes [26], and previous studies have reported that the degree of browning depends on the strain used in fermentation, including soy sauce [27] and soybean paste [28].

Hydrolytic enzymes from microorganisms play an important role in soybean fermentation because they are involved in the production of reducing sugars, amino acids, and their derivatives [29]. Especially, *A. oryzae* is well-known for producing high levels of hydrolytic enzymes, including amylase and protease [30]. α-Amylase and protease activities of moromi fermented with different *A. oryzae* strains are presented in Table 1. The α-amylase activities of moromi-1 (1046.78 unit/g) and moromi-2 (960.36 unit/g) were slightly higher than that of control moromi (952.57 unit/g), while the protease activity of moromi-2 (1237.15 unit/g) was higher than that of control moromi (1192.53 unit/g) and moromi-1 (1022.75 unit/g). Similarly, it was reported that the enzyme activities, including α-amylase, peptidase, and protease, differed depending on *A. oryzae* strains in rice koji [19] and soybean koji [21]. The hydrolytic enzyme activity of moromi is closely related to the specificity of *A. oryzae* strains, depending on the gene expression patterns of enzymes [13]. 

### 2.3. Sensory Evaluation

**Figure 2:**
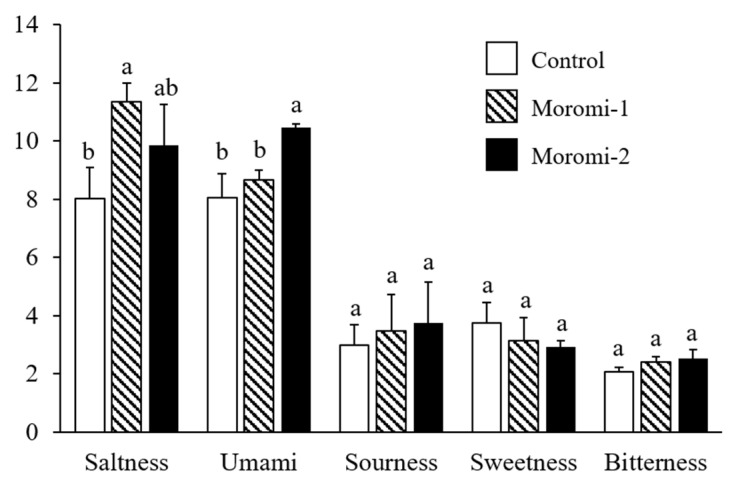
Quantitative descriptive sensory analysis of moromi samples prepared with different *A. oryzae* strains. Control, moromi-1, and moromi-2 are moromi samples fermented by commercial strain, *A. oryzae* KCCM13012P, and *A. oryzae* KCCM12804P, respectively. The intensity of each sensory quality was rated on a 15 cm line scale, labeled “very weak” and “very strong”, with 0.5 cm anchors on the left and right sides. For each taste, the different letters (a and b) in each bar indicate significant difference according to Duncan’s multiple test (*p* < 0.05).

The tastes of moromi samples fermented with different *A. oryzae* strains were evaluated (Figure 2). Three *A. oryzae* strains did not contribute to the differences in sourness, sweetness, and bitterness of moromi samples. However, moromi-1 and moromi-2 samples showed a saltier taste than a commercial moromi. In addition, the umami taste of moromi-2 was stronger than that of the other moromi samples. Saltiness and umami are mostly determined tastes in fermented soy products, including soy sauce [31,32] and soybean paste [33], and these tastes are related to metabolite profiles formed by microbial metabolism [31,32]. Many previous studies confirmed that the small metabolites, including sugars, acidic compounds, amino acids, and peptides produced during fermentation, contributed to the sensory qualities of fermented foods, such as soy sauce [34] and soybean paste [35]. To examine the correlation between moromi metabolites and sensory quality, the metabolite profiles of moromi samples fermented with different *A. oryzae* strains were analyzed.

### 2.4. Metabolomic Analysis

**Figure 3:**
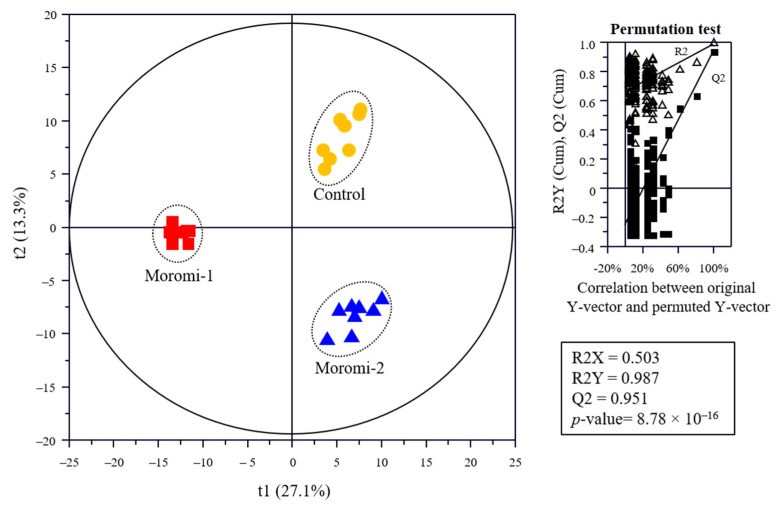
Partial least-squares discriminant analysis (PLS-DA) score plot of metabolites of the moromi fermented with different *A. oryzae* strains and its quality parameters. Control, moromi-1, and moromi-2 are moromi samples fermented by commercial strain, *A. oryzae* KCCM13012P, and *A. oryzae* KCCM12804P, respectively. Metabolites were analyzed using GC-MS, UPLC-Q-TOF MS, amino acid analyzer, and HPLC. The qualification of the PLS-DA model was evaluated using R2X, R2Y, Q2, and *p*-value and validated using cross validation with a permutation test (*n* = 200).

**Figure 4:**
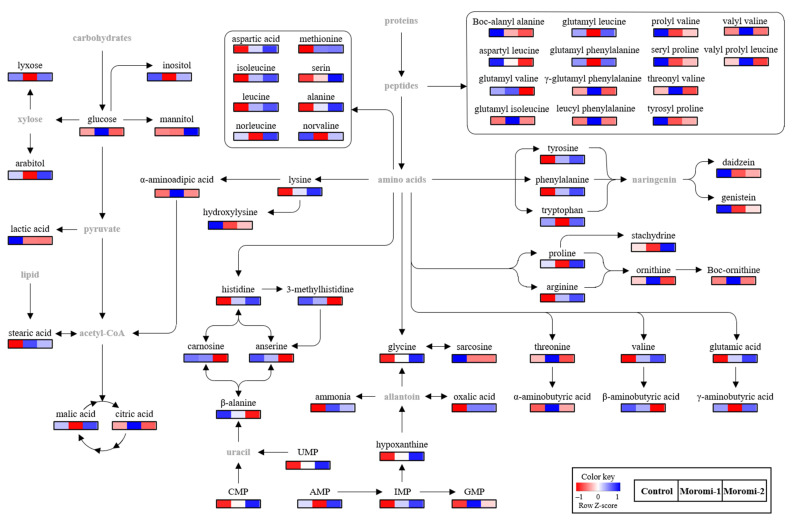
Proposed pathway related to *A*. *oryzae* fermentation. Control, moromi-1, and moromi-2 are moromi samples fermented by commercial strain, *A. oryzae* KCCM13012P, and *A. oryzae* KCCM12804P, respectively. The heat maps showed the relative abundance of identified metabolites. Blue and red indicate the increase and decrease in metabolite levels, respectively. CMP, cytidine monophosphate; UMP, uridine monophosphate; GMP, guanosine monophosphate; IMP, inosine monophosphate; AMP, adenosine monophosphate.

The metabolite profiles in moromi samples fermented with different *A. oryzae* strains were analyzed using an amino acid analyzer, high performance liquid chromatography (HPLC), gas chromatography (GC)-mass spectrometry (MS), and ultra-high-performance liquid chromatography-quadrupole-time-of-flight (UPLC-Q-TOF) MS, and based on metabolite profiles, the discrimination of moromi samples was visualized using the Partial least-squares discriminant analysis (PLS-DA) score plot (Figure 3). The goodness of fit (R2X = 0.503; R2Y = 0.987), predictability (Q2 = 0.951), *p*-value (8.78 × 10^−7^), and the cross-validation (y intercepts of R2 < 0.5 and Q2 < −0.2) determined by the permutation test (n = 200) indicated that the PLS-DA model was statistically acceptable (Figure 3). In the PLS-DA score plot, three moromi samples were clearly separated from each other by t(1) and t(2). The VIP and *p*-values of individual metabolites were analyzed to find metabolites that contributed to the difference between moromi samples (Table 2 and Tables S1–S3). Sixty-six metabolites, including 30 amino acids, 16 peptides, 6 nucleotides, 5 sugars, 4 acidic compounds, 2 lipids, 1 isoflavones, and ammonia were identified, and their VIP and *p*-values were VIP ≥ 0.83 and *p*-value < 0.05, respectively. Similar metabolite profiles were observed in previous studies on the fermentation of soybean by *A. oryzae* KACC 46102 [18] and *A. oryzae* KCTC6983 [36]. In addition, the normalized chromatogram intensities between moromi samples fermented with different *A. oryzae* strains were compared (Figure 4), and their fold changes were calculated against the commercial strain (Table 2). 

### 2.5. Amino Acids and Peptides

In identified metabolites, amino acids and peptides were the major metabolites in moromi, and their profiles were significantly different according to the *A. oryzae* strains. The newly isolated strains increased the production of most major amino acids, including aspartic acid, glycine, serine, isoleucine, glutamic acid, alanine, valine, lysine, and arginine, compared with the control, and in particular, their contents in moromi-2 were two times more than those of control moromi, while the contents of glycine, isoleucine, and threonine of moromi-1 were more than two times higher than those of control moromi. In particular, the level of Boc-ornithine in moromi-1 was about 63 times higher than those of control moromi. However, some minor amino acids, including sarcosine, β-methylhistidine, hydroxylysine, β-alanine, and β-aminobutyric acid, were decreased in moromi-1 and moromi-2. The changes in these amino acids play a crucial role in the taste of fermented soybean foods [34,35]. Especially, aspartic acid and glutamic acid mainly contribute to the umami taste [32], and moromi-2, which showed the highest umami intensity, had the highest content of aspartic acid and glutamic acid (Table S1). In addition, umami amino acids and some amino acids, including glycine, lysine, arginine, histidine, and ornithine, can act as salt taste enhancers [37]. 

Like amino acids, the levels of moromi peptides were significantly changed according to *A. oryzae* strains. Compared with the control moromi, the levels of carnosine, glutamyl valine, aspartyl leucine, Boc-alanyl alanine, valyl valine, γ-glutamyl phenylalanine, threonyl valine, valyl prolyl leucine, and leucyl phenylalanine were lower by two times in moromi-2, whereas the level of glutamyl isoleucine was higher by about two times. Unlike moromi-2, the levels of seryl proline, Boc-alanyl alanine, prolyl valine, tyrosyl proline, glutamyl leucine, and glutamyl phenylalanine decreased in moromi-1, whereas other identified peptides, except for glutamyl valine, were significantly increased. In particular, the levels of glutamyl isoleucine and valyl valine in moromi-1 were about 30 and 15 times higher than those of control moromi. These peptides provide the taste background and enhance other tastes [31]. Some small peptides, such as glutamic-acid-containing peptides, also exhibit an umami taste [31], and peptides containing bitter amino acids, such as valine, leucine, and phenylalanine, exhibit bitterness [31,38]. In this study, moromi-2 showed the most intense umami taste, but there was no difference in bitterness between moromi samples (Figure 2). As for soy sauce, the direct contribution of peptides to its taste is negligible due to the relatively low content [31]. In addition, some amino acids (arginine, aspartic acid, and glutamic acid) and salt can act as bitterness suppressors [39,40]. 

### 2.6. Nucleotides

In addition to amino acids, nucleotides, known as enhancers of umami taste [32], were also significantly altered depending on the *A. oryzae* strains (Table 2). Among nucleotides, AMP was the major nucleotide, and its concentrations in control and moromi-2 were 2.8 and 3.9 times higher than those of moromi-1. The concentrations of other nucleotides in the moromi samples fermented with newly isolated strains were higher than those of control moromi. In particular, the CMP contents in moromi-1 and moromi-2 samples were 3.62 and 6.54 times, respectively, higher than those of control moromi, and their GMP, which were well-known umami enhancers along with IMP [32], were 6.61 and 3.02 times higher, respectively. UMP, which was not detected in control moromi, was newly produced in moromi fermented with newly isolated strains. The high content of these nucleotides in moromi-2, which had higher umami than monosodium glutamate [41], might positively contribute to an increase in the umami taste in moromi-2. 

### 2.7. Other Metabolites and Metabolomic Pathway

The profiles of minor metabolites, including sugars, acidic compounds, lipids, and secondary metabolites, were also significantly changed according to *A. oryzae* strains (Table 2). Among them, sugar alcohols, including mannitol and arabitol, increased in moromi-2 but decreased in moromi-1, except for glucose. In addition, lactic acid, which contributes to sourness or sour aroma in soy sauce [42], was about three times lower in moromi-1 and moromi-2 samples than that of control moromi. Moreover, the levels of daidzein and genistein, which are major bioactive compounds associated with the health benefits of soy products [43], were also decreased in moromi-1 and moromi-2 samples. 

Based on the identified metabolites, the metabolomic pathway associated with moromi fermentation by different *A. oryzae* strains was proposed (Figure 4). The amino acid and peptide metabolisms were mainly changed during moromi fermentation, while the energy, nucleotide, and isoflavone metabolisms were also observed. Similar metabolisms were also reported in previous metabolomics studies on the fermentation of soy sauce with *A. oryzae* 3.042 and 100-8 [44], and soybean residue with *A. oryzae* KCTC6983 [36].

### 2.8. Correlation Analysis

**Figure 5:**
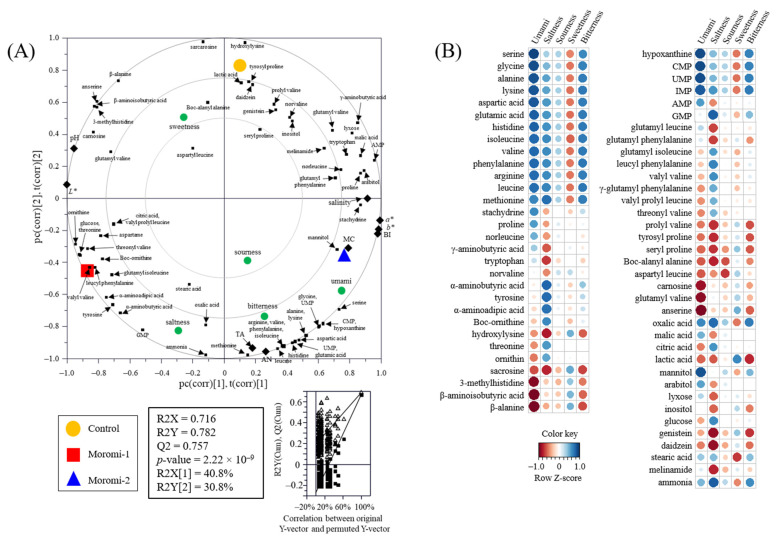
Partial least-squares discriminant analysis (PLS-DA) biplot (**A**) and Pearson correlation between sensory quality and metabolites (**B**). Control, moromi-1, and moromi-2 are moromi samples fermented by commercial strain, *A. oryzae* KCCM13012P, and *A. oryzae* KCCM12804P, respectively. PLS-DA biplot was drawn based on the general quality characteristics, sensory quality, and metabolite profiles. The statistical acceptability was evaluated by quality parameters (R2X, R2Y, Q2, and *p*-value) and validated by cross validation with a permutation test (*n* = 200). Correlation heat map colors represent the correlation coefficients, and blue and red colors on a red–blue color scale indicate the positive and negative correlations, respectively. L*, lightness; a*, redness; b*, yellowness; BI, browning index; TA, total acidity; MC, moisture content; AN, amino type nitrogen; CMP, cytidine monophosphate; UMP, uridine monophosphate; GMP, guanosine monophosphate; IMP, inosine monophosphate; AMP, adenosine monophosphate.

The relationship between metabolite profiles, general characteristics, and sensory quality was visualized using a PLS-DA biplot with acceptable parameters, including the goodness of fit (R2X = 0.716; R2Y = 0.782), predictability (Q2 = 0.757), *p*-values (2.22 × 10^−9^), and permutation test (n = 200) (Figure 5A). Metabolite profiles, general characteristics, and sensory characteristics were significantly separated by t(1) and t(2) according to *A. oryzae* strains. Moreover, Pearson’s correlation coefficient between metabolite profiles and sensory characteristics was calculated and visualized using heatmaps (Figure 5B and Table S4). In particular, umami taste had strong positive correlations (r > 0.83) with nucleotides, including hypoxanthine, CMP, UMP, and IMP, and with some amino acids, including serine, glycine, alanine, lysine, aspartic acid, glutamic acid, histidine, isoleucine, valine, phenylalanine, arginine, and leucine (r > 0.76). In contrast, some amino acid derivatives, such as 3-methylhistidine, β-aminoisobutyric acid, and β-alanine, and some peptides, such as carnosine, glutamyl valine, and anserine, showed negative correlations with umami taste (r < −0.85). In saltiness, methionine, tyrosine, α-aminobutyric acid, α-aminoadipic acid, GMP, oxalic acid, and ammonia showed positive correlations (r > 0.74), whereas tryptophan, hydroxylysine, sacrosine, prolyl valine, tyrosyl proline, seryl proline, Boc-alanyl alanine, isoflavones, and melinamide showed negative correlations (r < −0.70). The strong correlations between amino acids or peptides and taste (umami and salty) were also observed in various fermented foods, including fermented soybean [45], soybean paste [35], and moromi extract [46]. These results indicate that moromi qualities were strongly associated with the change in metabolite profiles produced by *A. oryzae* strains.

## 3. Materials and Methods

### 3.1. Isolation and Identification of A. oryzae

Commercialized strain (*A. oryzae* KACC47838) was purchased from Chungmoo fermentation (Ulsan, Korea), and traditional soybean paste for isolation of strains was purchased from the local market (Sunchang, Korea). To isolate *A. oryzae* strains, 10 g of soybean paste was homogenized using 90 mL of pasteurized saline (0.85%, *w*/*v*). Following centrifugation at 4000 rpm for 10 min, the diluted supernatant was plated into potato dextrose agar (PDA, Difco, Detroit, MI, USA), malt extract agar (MEA, Difco), and yeast peptone dextrose agar (YPD, Difco), respectively. Following incubation for seven days at 30 °C, the spore was obtained, and the spore solution diluted using pasteurized salinity water (0.85%, *w*/*v*) was plated into a PDA agar. After incubation at 30 °C for seven days, two spores were isolated and stored at 4 °C until used.

The identification of isolated mold from traditional fermented foods was performed using the sequencing of the internal transcribed spacer (ITS) region. PCR amplification was conducted using the ITS1 (5′-TCCGTAGGTGAACCTGCGG-3′) and ITS4 (5′-TCCTCCGCTTATTGATATGC-3′) primers for the ITS gene, and the gene sequencing data were identified by the National Center for Biotechnology (NCBI) nucleotide BLAST. Homology with other strains was analyzed using registered strains in NCBI (https://blast.ncbi.nlm.nih.gov/Blast.cgi (accessed on 10 June 2021). The phylogenetic tree was established using MEGA-X software (version 10.2.1) based on their nucleotide sequence. The maximum likelihood method was used for evolutionary distance inference, and 1000 bootstrap analysis was performed [47].

### 3.2. Moromi Fermentation

Soybean soaked at 25 °C for 18 h was steamed at 121 °C for 30 min, and then each isolated strain sub-cultured in yeast mold agar (YM, Difco) and a commercial strain (control), respectively, was inoculated with 10^7^~10^8^ CFU/mL to the soybean. The inoculated soybean was fermented in three stages. Primary fermentation was performed at 30 °C for 48 h. After crushing the primary fermented soybean and adding 1.5 times water, secondary fermentation was performed at 10 °C for 48 h. Tertiary fermentation was carried out at 55 °C for 48 h after the addition of salt to the fermented mixture to a final salinity of 8%. Following three-step fermentation, the fermented samples were aged at room temperature for three weeks. The final products were filtrated using a cotton filter, freeze-dried, and stored at –18 °C [48]

### 3.3. Physicochemical Properties of Moromi

The moisture content was measured using an infrared moisture meter (JP/FD-720, Kett engineering, Tokyo, Japan). The pH, acidity, and amino acid nitrogen were measured using an automatic titrator T50 (Mettler-Toledo GmbH, Greifensee, Switzerland). The salinity was measured using a digital salinity meter (TM-30D, Takemura Electric works Ltd., Tokyo, Japan), and the color was measured using a color meter (Konica Minolta Sensing, Inc., Osaka, Japan).

### 3.4. Enzymatic Activities of Moromi

Enzyme activities, including α-amylase and protease of the three different *A. oryzae* strains were evaluated using samples after primary fermentation. The α-amylase activity of samples was measured following the method described in Gupta et al. (2003) [49] with a minor modification. Sample (100 μL) was mixed with 200 μL of 1% starch solution and incubated at 40 °C for 30 min. After stopping the reaction with the addition of 300 μL of 1 N HCl, 800 μL of Lugol’s solution was added, and the absorbance of starch remaining in the solution was measured at 660 nm. The yield of converted glucose was calculated from degraded starch, and one unit (U) of the α-amylase activity was defined as the amount of enzyme releasing glucose (mg) from starch by 1 g of sample for 1 min. Protease activity of samples was measured by a Folin and Ciocalteu’s reagent method [50] with a minor modification. Sample (200 μL) was mixed with 500 μL of 0.65% casein solution in 50 mM sodium phosphate buffer (pH 7.5) and incubated at 37 °C for 10 min. After stopping the reaction by adding 500 μL of trichloroacetic acid for 30 min at 37 °C, the reaction mixture was centrifuged at 10,000× *g* for 3 min at 4 °C, and the supernatant (200 μL) mixed with 200 μL of Na_2_CO_3_ and 100 μL of 0.5 M Folin and Ciocalteu’s reagent was incubated at 37 °C for 30 min. Following centrifugation at 10,000× *g* for 3 min, the absorbance of the supernatant was measured at 660 nm. Tyrosine was used as a standard compound, and one U of the protease activity was defined as the amount of enzyme releasing 1 μg of tyrosine from casein by 1 g of sample for 1 min.

### 3.5. Sensory Evaluation

Sensory analysis was carried out by eight trained panelists aged 21–24 years, and all panelists were trained for at least three months. Before sensory analysis, the panelists discussed a series of taste reference solutions, including sodium chloride (NaCl) (3%) for saltiness, monosodium glutamate (MSG) (0.3%) for umami taste, citric acid (0.2%) for sourness, sucrose (2%) for sweetness, and quinine (0.0025%) for bitterness. The intensity of each sensory quality was scored on a 15-cm-line scale with 0.5 cm anchors on the left and right end sides labeled “very weak” and “very strong” [33].

### 3.6. Free Amino Acid Analysis

To extract free amino acids, a lyophilized sample (2 g) was homogenized with distilled water under sonication for 20 min. Following centrifugation, 2 mL of the supernatant was mixed with 2 mL of 5% trichloroacetic acid and sequentially filtrated using a 0.2 μm syringe filter. The 20 μL of the filtrated solution was injected into the amino acid analyzer (Hitachi L-8900, Hitachi High-Technologies, Tokyo, Japan). For the separation and detection of amino acids, a Hitachi ion exchange column (4.6 mm × 60 mm, Hitachi High-Technologies) with an ammonia filtering column (4.6 mm × 40 mm, Hitachi High-Technologies) was used to separate amino acids, the mobile phase used as a wide range of pH buffer set (pH-Set, Kanto Chemical Co. Inc., Tokyo, Japan), and UV/Vis (440–570 nm) was used as the detector. The column temperature, flow rate, and ninhydrin flow rate were set at 50 °C, 0.4 mL/min, and 0.35 mL/min, respectively [51]. 

### 3.7. Nucleotide Analysis

To extract nucleotides, a lyophilized sample (5 g) was homogenized using 20 mL of aqueous perchloric acid and filtrated using a filter paper (Whatman No. 1). The pH of the extract was adjusted to 5.5 using the addition of 1 M KOH and to the volume of 50 mL using 0.6 M perchloric acid (pH 5.5). Following filtration, the sample was analyzed using HPLC (Agilent 1200 series, Agilent Technologies, Santa Clara, CA, USA) coupled with the CAPCELL PAK C18 column (250 mm × 4.6 mm, 5 μm, Shiseido, Tokyo, Japan). Next, 20 mM sodium phosphate buffer (pH 5.5) was used as a mobile phase with a flow rate of 1 mL/min. The column temperature was set at 30 °C, and nucleotide was detected at 254 nm [52]. 

### 3.8. Metabolomic Analysis

#### 3.8.1. GC/MS-Based Metabolite Analysis

The GC/MS-based metabolite analysis was performed with some modifications to a previous method [46]. To extract metabolites, the lyophilized sample was homogenized with 70% methanol containing dicyclohexyl phthalate as an internal standard (IS) using a bullet blender (Next Advance, Troy, NY, USA). The extracts were completely dried using a CentriVap SpeedVac concentrator (Labconco Co., Kansas City, MO, USA). The dried residues were dissolved in 100 µL of pyridine containing methoxyamine hydrochloride and incubated for 90 min at 37 °C. The methoxylated samples were then derivatized by adding 100 μL of N, O-bis(trimethylsilyl)trifluoroacetamide (BSTFA) for 30 min at 70 °C. The derivatized non-volatile metabolites were analyzed using a GC/MS system (Shimadzu Corp., Kyoto, Japan) equipped with a DB-5MS column (30 m × 0.25 mm, 0.25 μm film thickness, Agilent Technologies, Santa Clara, CA, USA) at a split ratio of 1:50. The injector temperature was set at 200 °C, and helium was used as the carrier gas at a flow rate of 0.95 mL/min. The oven temperature program was set as holding at 70 °C for 2 min, increasing from 70 to 320 °C at a rate of 10 °C/min, and holding 320 °C for 5 min. The effluent was detected using a GCMS-TQ 8030 MS (Shimadzu Corp., Kyoto, Japan) system with electron ionization at 70 eV. The ion source and interface temperatures were 230 and 280 °C, respectively. Data were monitored and collected in the full-scan mode in the *m*/*z* range from 45 to 550.

#### 3.8.2. UPLC-Q-TOF MS-Based Metabolite Analysis

UPLC-Q-TOF MS-based metabolite analysis was performed with some modifications to the previous method [46]. To extract metabolites, the lyophilized sample was homogenized with 70% methanol containing terfenadine (IS) using a bullet blender. Following centrifugation, the supernatants were analyzed by UPLC-Q-TOF MS (Waters Corp., Milford, MA, USA). The metabolites were separated using an Acquity BEH C18 column (2.1 mm × 100 mm, 1.7 µm; Waters, Milford, MA, USA) equilibrated with mobile phase A (water containing 0.1% formic acid). The metabolites were eluted by a linear gradient from 0% mobile phase B (acetonitrile containing 0.1% formic acid) to 100% B for 9 min. The eluted metabolites were detected using Q-TOF MS with positive electrospray ionization (ESI) mode. The desolvation temperature and flow rate were 400 °C and 800 L/h, respectively, and the source temperature was 120 °C. Sampling cone and capillary voltages were 40 V and 3 kV, respectively. Leucine-enkephalin ([M + H] = 556.2771) was used as the lock mass with an infusion flow rate of 20 µL/min. MS data were obtained with a scan range of 50 to 1500 *m*/*z*. 

#### 3.8.3. Data Processing

The GC/MS data were aligned with a retention time window of 0.1–0.05 min and normalized with the IS. Metabolites were identified using NIST 11 and Wiley 9 mass spectral libraries, and retention indices (RI). LC/MS data were collected using a peak-width at 5% height of 1s, peak-to-peak baseline noise of 1, noise elimination of 6, and an intensity threshold of 5000. The collected data were aligned with a 0.05 Da mass window and a retention time window of 0.2 min using the MarkerLynx software (version 4.1, Waters, Milford, MA, USA) and were normalized with the IS. Furthermore, metabolites were identified using the UNIFI software (version 1.9.2, Waters, Milford, MA, USA) connected to various online databases and the METLIN database.

### 3.9. Statistical Analysis

Multivariate statistical analysis was carried out using SIMCA-P+ v.16.0.1 (Umetrics, Umea, Sweden) and all variables were automatically transformed and scaled to unit variance. PLS-DA was used to visualize the differences in sample groups and correlations between the metabolites and general characteristics, including color, pH, total acidity, salinity, moisture contents, amino type nitrogen, enzymatic activities, and sensory evaluation. Statistical differences between the experimental data were statistically analyzed using one-way analysis of variance (ANOVA) with Duncan’s test using SPSS v.24.0 (SPSS Inc., Chicago, IL, USA). Pearson’s correlation coefficients between the sensory qualities and metabolites were calculated and visualized using the R software.

## 4. Conclusions

This study investigated the effect of newly isolated strains (*A. oryzae* KCCM12012P and KCCM12804P) on the quality of moromi using metabolomics, sensory evaluation, enzyme activity, and physicochemical properties and compared them with those of a commercial strain (control). Moromi samples prepared with different strains showed significant differences in physicochemical properties, enzyme activity, sensory quality, and metabolite profiles. In particular, the correlation data indicate that the major moromi metabolites, including amino acids, peptides, and nucleotides, contributed to the saltiness and umami taste of moromi samples. Moreover, the metabolomic pathway related to moromi fermentation according to different *A. oryzae* strains was proposed. Although volatile compounds contributing to moromi flavor quality and the mechanism of metabolite production according to *A. oryzae* strains were not investigated, the proposed results suggest that the quality of moromi fermented with isolated strains, especially *A. oryzae* KCCM12804P, was superior to that of a commercial strain. Therefore, these strains could be used as ideal strains to produce moromi and soy sauce with good sensory quality in the fermentation industry.

## Figures and Tables

**Table 1:** Physiochemical characteristics and enzymatic activities of the moromi fermented with different *Aspergillus oryzae* strains.

		Control	Moromi-1	Moromi-2
Physiochemical characteristics	appearance of moromi	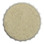	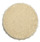	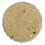
	L*	82.07 ^b^	89.00 ^a^	68.38 ^c^
	a*	2.16 ^a^	0.29 ^c^	6.89 ^a^
	b*	13.48 ^b^	11.75 ^c^	19.19 ^a^
	BI	19.50 ^b^	14.07 ^c^	39.81 ^a^
	pH	6.41 ^a^	6.44 ^a^	6.17 ^b^
	total acidity (%)	2.08 ^b^	2.39 ^a^	2.41 ^a^
	salinity (%)	8.00 ^a^	8.10 ^a^	8.00 ^a^
	moisture contents (%)	11.25 ^a^	11.22 ^a^	11.78 ^a^
	amino type nitrogen (mg %)	1175.11 ^c^	2061.93 ^b^	2221.47 ^a^
Enzymatic activity (unit/g)	α-amylase	952.57 ^c^	1046.78 ^a^	960.36 ^b^
	protease	1192.53 ^b^	1022.75 ^c^	1273.15 ^a^

Control, moromi-1, and moromi-2 are moromi samples fermented by commercial strain, *A. oryzae* KCCM13012P, and *A. oryzae* KCCM12804P, respectively. Different capital letters (a, b, and c) in the same row indicate a significant difference according to Duncan’s multiple test (*p* < 0.05). Browning index (BI) of samples was calculated by following equation: BI = [100(x − 0.31)]/0.17, where x = (a* + 1.75L*)/(5.645L* + a* − 3.012b*).

**Table 2:** Identification of major metabolites contributing to the separation of samples on the PLS-DA score plots and their fold change.

	Compounds	VIP	*p*-Value	Fold Change (vs. Control)	
				Moromi-1	Moromi-2
amino acids	glycine	1.68	1.46 × 10^−9^	2.38	3.73
	serine	1.67	1.32 × 10^−11^	1.68	2.94
	isoleucine	1.67	4.30 × 10^−7^	2.13	2.53
	aspartic acid	1.68	2.17 × 10^−9^	1.96	2.49
	glutamic acid	1.68	4.59 × 10^−11^	1.99	2.49
	stachydrine	1.28	1.21 × 10^−6^	UC	2.46
	alanine	1.68	7.10 × 10^−9^	1.85	2.44
	valine	1.67	3.86 × 10^−10^	1.92	2.25
	lysine	1.68	2.06 × 10^−9^	1.72	2.22
	arginine	1.67	8.84 × 10^−8^	1.86	2.16
	ammonia	1.54	1.62 × 10^−9^	2.49	2.10
	histidine	1.68	1.20 × 10^−8^	1.49	1.72
	leucine	1.66	4.32 × 10^−8^	1.50	1.65
	methionine	1.62	1.33 × 10^−8^	1.59	1.61
	phenylalanine	1.67	5.87 × 10^−8^	1.41	1.56
	proline	1.37	4.44 × 10^−13^	−2.91	1.43
	norleucine	1.20	1.61 × 10^−6^	−1.58	1.20
	tyrosine	1.33	6.89 × 10^−8^	1.53	1.07
	tryptophan	1.05	1.58 × 10^−4^	−2.22	1.10
	α-aminoadipic acid	1.32	1.21 × 10^−8^	1.58	1.04
	α-aminobutyric acid	1.33	2.92 × 10^−6^	1.20	1.04
	γ-aminobutyric acid	1.32	1.37 × 10^−7^	−1.60	−0.94
	norvaline	0.89	5.17 × 10^−3^	−2.14	−1.18
	ornithine	1.35	5.41 × 10^−9^	1.68	−1.51
	hydroxylysine	1.52	2.48 × 10^−7^	−1.98	−1.55
	β-alanine	1.65	2.49 × 10^−5^	−1.23	−1.83
	threonine	1.34	1.36 × 10^−9^	2.33	−1.86
	β-aminoisobutyric acid	1.64	2.02 × 10^−7^	−1.19	−4.19
	Boc-ornithine	1.19	4.94 × 10^−7^	63.28	−7.63
	β-methylhistidine	1.62	1.99 × 10^−5^	−1.27	UC
	sarcosine	1.62	5.46 × 10^−8^	UC	UC
peptides	glutamyl isoleucine	1.11	1.79 × 10^−5^	29.58	2.30
	glutamyl leucine	1.06	9.07 × 10^−5^	−7.69	1.18
	glutamyl phenylalanine	0.93	1.72 × 10^−3^	−4.89	1.09
	tyrosyl proline	1.00	8.92 × 10^−3^	−2.31	−1.80
	anserine	1.65	6.10 × 10^−9^	−1.13	−1.88
	seryl proline	0.88	2.96 × 10^−2^	−2.75	−1.88
	prolyl valine	1.11	7.87 × 10^−4^	−3.30	−1.92
	carnosine	1.52	9.23 × 10^−4^	−1.03	−2.14
	aspartyl leucine	0.94	2.77 × 10^−2^	−1.42	−2.26
	glutamyl valine	1.27	2.46 × 10^−5^	1.17	−5.22
	γ-glutamyl phenylalanine	1.11	1.80 × 10^−5^	4.59	−6.03
	Boc-alanyl alanine	1.09	5.09 × 10^−3^	−3.22	−8.84
	threonyl valine	1.27	5.85 × 10^−9^	3.41	−9.72
	valyl prolyl leucine	0.98	9.19 × 10^−4^	2.79	−9.98
	valyl valine	1.29	2.27 × 10^−10^	15.37	−10.26
	leucyl phenylalanine	1.28	8.30 × 10^−10^	+	UC
nucleotides	CMP	1.68	1.25 × 10^−13^	3.62	6.54
	GMP	1.39	2.65 × 10^−16^	6.61	3.02
	AMP	1.36	7.04 × 10^−16^	−2.85	1.39
	IMP	1.68	2.77 × 10^−8^	1.25	1.38
	hypoxanthine	1.68	2.91 × 10^−12^	1.13	1.26
	UMP	1.68	1.80 × 10^−12^	UC	UC
sugars	mannitol	1.58	1.62 × 10^−16^	−1.09	3.03
	arabitol	1.36	4.40 × 10^−13^	−6.42	1.43
	lyxose	1.33	8.18 × 10^−13^	−3.03	1.04
	inositol	0.98	1.14 × 10^−3^	−1.33	−1.06
	glucose	1.37	5.94 × 10^−22^	2.81	−1.54
acidic compounds	malic acid	1.35	1.94 × 10^−13^	UC	1.40
	citric acid	0.96	9.17 × 10^−4^	1.44	−1.11
	lactic acid	1.59	3.92 × 10^−16^	−3.07	−3.12
	oxalic acid	1.67	1.41 × 10^−18^	+	+
lipids	stearic acid	0.83	4.00 × 10^−2^	1.10	1.07
	melinamide	0.96	1.43 × 10^−3^	UC	−1.33
isoflavones	genistein	1.01	3.37 × 10^−3^	−3.58	−1.79
	daidzein	1.18	4.46 × 10^−4^	−3.53	−2.31

Control, moromi-1, and moromi-2 are moromi samples fermented by commercial strain, *A. oryzae* KCCM13012P, and *A. oryzae* KCCM12804P, respectively. *p*-values were analyzed by Duncan’s test. VIP, variable importance in the projection; UC, uncalculated; +, newly generated; CMP, cytidine monophosphate; UMP, uridine monophosphate; GMP, guanosine monophosphate; IMP, inosine monophosphate; AMP, adenosine monophosphate.

## Data Availability

Data are contained within the article and Supplementary Materials.

## Supplementary Materials

The following supporting information can be downloaded at: https://www.mdpi.com/article/10.3390/molecules27196182/s1, Figure S1: Result of internal transcribed spacer (ITS) sequencing of isolated *A. oryzae* strains; Table S1: Quantitative analysis of amino acids and nucleotides; Table S2: Identification of major metabolites contributing to the separation of samples on the PLS-DA score plots by GC/MS analysis; Table S3: Identification of major metabolites contributing to the separation of samples on the PLS-DA score plots by UPLC-Q-TOF MS analysis; Table S4: Pearson correlation coefficient between sensory characteristics and metabolite profiles.

## Sample Availability

Samples of the strains are available from the Microbial Institute for Fermentation Industry.
