# Supplementary material for: Comparative Metabolomic Analysis of Moromi Fermented Using Different Aspergillus oryzae Strains
Source: Molecules. 2022 Sep 21;27(19):6182. doi: 10.3390/molecules27196182 (PMC9573031; doi:10.3390/molecules27196182)
Supplement: Supplementary file 1 [file molecules-27-06182-s001.zip › molecules-1888402-supplementary.pdf]

KCCM13012P(ITS1)

GTTTTTTGGGTGTAGGATCTAGCGAGCCCACCTCCCACCCGTGTTTACTGTACCTTAGTTGCTTCGGCGGGC
CCGCCATTCATGGCCGCCGGGGGCTCTCAGCCCCGGGCCCGCGCCCGCCGGAGACACCACGAACTCTGTCT
GATCTAGTGAAGTCTGAGTTGATTGTATCGCAATCAGTTAAAACTTTCAACAATGGATCTCTTGGTTCCGG
CATCGATGAAGAACGCAGCGAAATGCGATAACTAGTGTGAATTGCAGAATTCCGTGAATCATCGAGTCTT
TGAACGCACATTGCGCCCCCTGGTATTCCGGGGGGCATGCCTGTCCGAGCGTCATTGCTGCCCATCAAGCA
CGGCTTGTGTGTTGGGTCGTCGTCCCCTCTCCGGGGGGGACGGGCCCCAAAGGCAGCGGCGGCACCGCGT
CCGATCCTCGAGCGTATGGGGCTTTGTCACCCGCTCTGTAGGCCCGGCCGGCGCTTGCCGAACGCAAATCA
ATCTTTTCCAGGTTGACCTCGGATCAGGTAGGGATACCCGCTGAACTTAAGCATATCATAAGCCCGGAGGA
AGATCATTACCGAGTGTAGGGTTCCTAGCGAGCCAACCTCCACGTGTTTACTGTACCTTAGTTGCTTCGGC
GGGCCCCTTTCGGGCCCCGGGGGCTCTGCCCGGGCCGCGCCGCCGGAGAACCCGAACTCTGTCTGATCTAG
TGAAAGTCTGAAGTTGAATTGGTATCGCAATCAGTTTAAAACTTTTCAACAATGGGATCTCTTGGGTTCCG
GCATCGATTAAAAAAACGCAGCGCAAATGCGAATAAACTAGGTGGTGAAATTTGCAAAAAATTCGGGGA
AATCATCGGAGTTCTTTTGAAACGCCACATTGGCCGCCCCGGGGTTATTTCCGGGGGGCGTGCTGGCCGGC
CTAAAAATGGTTTACTCGCCCCCCCTCTGGTTTATGGTTGTGTGCCCCCCCTGTCTCCGCCAGGAGACCTTA
CTGCCAGGGGGGGGGACAAGGCCCCACCACCAACCCCAAATTCAAAACGATGGTGTTGCTTCTACTTCCA
ACTTCTATGAGGGGGGGGGTTCGCACGGCACCCCTATAACATAAAAAAACCCTTATCCTCCTTTTCTTTTTT
TCAGACACTAGCAAACTTATATTATATACATTTTTTTTACCAAAAGATTTGAAAAACCAAATTTCCCGAA
AAAAGGGGTCAGCCAGGGCCAAACCCCCCGAAAAAAAAATTTTATAACCCTTTTCTACGAGAAAATAACA
GGGGAACGAAATAATATAATAACTCTTCCTCATTCCTGTTTACCTTCTACCTCCGTTTTCTTCTTATCTCCCT
TGTCTTTCCGCTGTTGCCCTGTTTCGCTATTCACCTTCCTCATTACCCGCTCTCTTATCTAATACCTCTATGC
CTCTACATAACTAGTCATAAATCACATTAAATTATAAA

KCCM12804P(ITS1)

GGGCCTCCTAGCGTAGGATCTAGCGAGCCCAACCTCCCACCCGTGTTTACTGTACCTTAGTTGCTTCGGCG
GGCCCGCCATTCATGGCCGCCGGGGGCTCTCAGCCCCGGGCCCGCGCCCGCCGGAGACACCACGAACTCT
GTCTGATCTAGTGAAGTCTGAGTTGATTGTATCGCAATCAGTTAAAACTTTCAACAATGGATCTCTTGGTTC
CGGCATCGATGAAGAACGCAGCGAAATGCGATAACTAGTGTGAATTGCAGAATTCCGTGAATCATCGAGT
CTTTGAACGCACATTGCGCCCCCTGGTATTCCGGGGGGCATGCCTGTCCGAGCGTCATTGCTGCCCATCAA
GCACGGCTTGTGTGTTGGGTCGTCGTCCCCTCTCCGGGGGGGACGGGCCCCAAAGGCAGCGGCGGCACCG
CGTCCGATCCTCGAGCGTATGGGGCTTTGTCACCCGCTCTGTAGGCCCGGCCGGCGCTTGCCGAACGCAAA
TCAATCTTTTTCCAGGTTGACCTCGGATCAGGTAGGGATACCCGCTGAACTTAAGCATATCAAAGACCGGG
AAGAAAATCATTACCGAGTGTAGGGGTCCTAGCGAGCCCAACCTCCCACCGTGGTTTACGGTACCTTAAGT
TGCTTCGGCGGGCCGCCGTTCAGGGCCGCGGGGGGCTCTAACCGGCGCGCCGCCGGAAACCCAGAAATCT
GTCTGATCTAGTGGAAGTCTGAGGTTGATGGGTATCGCATTCAGTTTAAAACTTTCAACAATGGGATCTTT
GGGGTTCCGGCATCGATTAAAAAAAGCAGCGGAAATGCGAATAACTAGGTGTGAAATTGCAGAAATTCCG
TGAAATCAACGAGGTCTTTTGAAACGCACATTGGCGCCCCCGGGGTATTTCCGGGGGGGGCATGGCCTGTT
CCAAACGGTCAATTGCTGCCCTTCAAGCAAGGGCCGGGGGTTGTTTGGGGGTCGCCGCTCCCCCCTCCCGG
GGGGGGGAAAGGGGCCACAAAAGGCCTCCGGCAGCCCCCCCCTCCCAATTCCTCCGCGGGGAAGGGGGG
GGCTTTTTGTTCACCCCCCTTTTTGTAAAAGCCGCGGGCCGCGGGCCTTTTGGGCGAGAAAGAAAAATAAA
AATTTTTTGTTTCCTCGTGGGAAAACCTCCAGAAAACAAGAGGATGGGGGGAAAACCGCCCCCTGAAAAA
TTTATAACCGATTAATAAAATTTGGTGGAGAGGAAGAAAAAAAAAAAAATATTTGTTGTTTCG

Figure S1. Result of internal transcribed spacer (ITS) sequencing of isolated *A. oryzae* strains.

Table S1. Quantitative analysis of amino acids and nucleotides.

	Compounds	Control	Moromi-1	Moromi-2
Amino acids (mg/kg)	glycine	3985.31[c]	9467.81[b]	14,879.67[a]
	serine	5406.10[c]	9085.63[b]	15,910.11[a]
	isoleucine	8036.75[c]	17,090.04[b]	20,359.86[a]
	aspartic acid	15,872.19[c]	31,033.80[b]	39,595.43[a]
	glutamic acid	16,986.61[c]	33,825.03[b]	42,325.51[a]
	alanine	11,871.07[c]	21,981.33[b]	28,910.20[a]
	valine	13,798.43[c]	26,472.91[b]	30,985.48[a]
	lysine	12,382.62[c]	21,302.60[b]	27,448.20[a]
	arginine	36,119.37[c]	67,077.16[b]	77,862.37[a]
	ammonia	3465.71[c]	8631.54[a]	7280.31[b]
	histidine	35,848.10[c]	53,491.70[b]	61,726.82[a]
	leucine	23,319.62[c]	35,026.16[b]	38,480.06[a]
	methionine	5642.84[b]	8984.42[a]	9065.42[a]
	phenylalanine	13,610.32[c]	19,188.11[b]	21,243.64[a]
	tyrosine	10,419.43[b]	15,917.88[a]	11,123.56[b]
	α-aminoadipic acid	4428.99[b]	6982.24[a]	4596.04[b]
	α-aminobutyric acid	1558.80[b]	1872.99[a]	1617.83[b]
	γ-aminobutyric acid	1045.29[a]	654.02[b]	1107.06[a]
	ornithine	4023.57[b]	6770.16[a]	2663.73[c]
	hydroxylysine	2387.02[a]	1207.53[c]	1541.30[b]
	β-alanine	583.40[a]	472.72[b]	318.63[c]
	threonine	2898.27[b]	6749.25[a]	1559.05[c]
	anserine	14,238.10[a]	12,580.54[b]	7557.45[c]
	carnosine	1093.98[a]	1059.85[a]	511.33[b]
	β-aminoisobutyric acid	3381.73[a]	2842.03[b]	806.90[c]
	β-methylhistidine	546.21[a]	428.42[b]	ND
	sarcosine	3651.98	ND	ND
Nucleotides (mg/kg)	CMP	34.90[c]	126.39[b]	228.35[a]
	GMP	94.42[c]	624.02[a]	284.83[b]
	AMP	1218.80[b]	428.32[c]	1694.55[a]
	IMP	25.19[c]	31.46[b]	34.66[a]
	hypoxanthine	172.16[c]	193.91[b]	217.62[a]
	UMP	ND	27.51[b]	54.37[a]

Control, moromi-1, and moromi-2 are moromi samples fermented by commercial strain, *A. oryzae* KCCM13012P, *and A. oryzae* KCCM12804P, respectively. ND, not detected; CMP, cytidine monophosphate; UMP, uridine monophosphate; GMP, guanosine monophosphate; IMP, inosine monophosphate; AMP, adenosine monophosphate. The different letters (a, b, and c) in the same row indicate significant differences according to Duncan's multiple test ($p < 0.05$).

Table S2. Identification of major metabolites contributing to the separation of samples on the PLS-DA score plots by GC/MS analysis.

RT (min).	Compound	RI
6.39	lactic acid	1098
7.74	oxalic acid	1136
8.95	norvaline	1211
9.82	norleucine	1267
10.24	proline	1294
12.89	malic acid	1474
15.09	lyxose	1652
15.78	arabitol	1709
16.93	citric acid	1809
17.78	glucose	1886
18.14	mannitol	1920
19.82	inositol	2083
21.32	stearic acid	2241

RT, retention time; RI: retention index.

Table S3. Identification of major metabolites contributing to the separation of samples on the PLS-DA score plots by UPLC-Q-TOF MS analysis.

RT	Compounds	Exact mass (m/z)	MS fragments
0.78	stachydrine	144.1019	70, 102
0.79	seryl proline	203.1046	116, 70, 125
1.07	glutamyl valine	247.1361	72, 217, 118
1.46	aspartyl leucine	247.1334	80
1.93	Boc-alanyl alanine	261.1464	136, 241
2.43	valyl valine	217.1538	116, 70, 130, 84
2.48	prolyl valine	215.1415	116, 70, 130
2.58	γ-glutamyl phenylalanine	295.1294	214, 130, 84
2.6	threonyl valine	219.1348	116, 70, 143
2.64	tyrosyl proline	279.1359	136, 116, 70, 262
2.67	Boc-ornithine	233.1489	70
2.72	glutamyl isoleucine	261.149	84, 86
2.86	tryptophan	205.0972	188,118, 144, 170
2.99	glutamyl leucine	261.1455	229, 86, 215, 201
3.10	glutamyl phenylalanine	295.1294	120, 84, 146, 188
3.18	valyl prolyl leucine	328.2231	215, 116, 70, 86
3.36	leucyl phenylalanine	279.1694	86, 120, 187
4.3	daidzein	255.0637	137
4.76	genistein	271.0582	153
8.82	melinamide	384.3265	105

RT, retention time.

Table S4. Pearson correlation coefficient between sensory characteristics and metabolite profiles.

	Umami	Saltness	Sourness	Sweetness	Bitterness
serine	0.91	0.34	0.31	−0.50	0.65
glycine	0.89	0.45	0.30	−0.53	0.67
alanine	0.86	0.54	0.33	−0.53	0.69
lysine	0.85	0.54	0.33	−0.54	0.71
aspartic acid	0.85	0.57	0.34	−0.53	0.70
glutamic acid	0.83	0.59	0.33	−0.55	0.71
histidine	0.81	0.61	0.34	−0.54	0.72
isoleucine	0.79	0.63	0.35	−0.58	0.70
valine	0.79	0.64	0.33	−0.55	0.71
phenylalanine	0.78	0.64	0.34	−0.53	0.72
arginine	0.78	0.64	0.33	−0.53	0.69
leucine	0.76	0.66	0.34	−0.54	0.73
methionine	0.66	0.76	0.33	−0.52	0.70
stachydrine	0.63	−0.37	0.00	−0.24	0.24
proline	0.57	−0.42	0.15	−0.06	0.07
norleucine	0.49	−0.44	0.12	0.02	−0.13
γ−aminobutyric acid	0.35	−0.61	0.02	0.10	−0.11
tryptophan	0.23	−0.70	−0.20	0.01	−0.23
norvaline	0.06	−0.48	0.22	0.26	−0.10
α−aminobutyric acid	−0.10	0.79	0.14	−0.23	0.40
tyrosine	−0.14	0.77	0.12	−0.18	0.28
α−aminoadipic acid	−0.20	0.74	0.09	−0.16	0.23
Boc−ornithine	−0.28	0.64	0.01	−0.29	0.12
hydroxylysine	−0.45	−0.80	−0.23	0.49	−0.61
threonine	−0.46	0.58	−0.02	0.01	0.01
ornithin	−0.52	0.53	−0.04	0.05	−0.02
sarcosine	−0.65	−0.76	−0.31	0.51	−0.68
3−methylhistidine	−0.91	−0.26	−0.34	0.39	−0.52
β−aminoisobutyric acid	−0.94	−0.20	−0.27	0.45	−0.59
β−alanine	−0.95	−0.38	−0.28	0.48	−0.65
Hypoxanthine	0.90	0.44	0.32	−0.54	0.68
CMP	0.90	0.43	0.31	−0.54	0.68
UMP	0.89	0.45	0.31	−0.54	0.68
IMP	0.83	0.59	0.33	−0.53	0.71
AMP	0.56	−0.48	0.08	−0.07	0.06
GMP	0.08	0.82	0.16	−0.32	0.43
glutamyl leucine	0.27	−0.68	−0.08	−0.06	−0.12
glutamyl phenylalanine	0.17	−0.69	−0.20	0.18	−0.44
glutamyl isoleucine	−0.25	0.57	−0.16	−0.35	0.14
leucyl phenylalanine	−0.26	0.66	0.03	−0.28	0.12
valyl valine	−0.33	0.62	−0.07	−0.23	0.12
aspartame	−0.44	0.50	−0.02	−0.28	−0.10
valyl prolyl leucine	−0.45	0.48	−0.04	−0.09	−0.12
threonyl valine	−0.49	0.46	−0.23	−0.09	0.02
prolyl valine	−0.52	−0.74	−0.28	0.29	−0.64
tyrosyl proline	−0.56	−0.75	−0.21	0.20	−0.65
seryl proline	−0.58	−0.72	−0.45	0.40	−0.62
Boc−alanyl alanine	−0.62	−0.78	−0.65	0.13	−0.49
aspartyl leucine	−0.62	−0.47	−0.71	0.32	−0.19
carnosine	−0.85	−0.14	−0.28	0.24	−0.42
glutamyl valine	−0.89	0.07	−0.21	0.31	−0.34
anserine	−0.93	−0.23	−0.27	0.49	−0.59
oxalic acid	0.65	0.77	0.37	−0.52	0.69
malic acid	0.48	−0.50	0.12	−0.01	0.02
citric acid	−0.51	0.59	0.09	−0.06	0.05
lactic acid	−0.59	−0.62	−0.12	0.65	−0.76
mannitol	0.87	0.03	0.28	−0.39	0.46
arabitol	0.53	−0.46	0.13	0.00	0.00

lyxose	0.29	−0.61	0.07	0.15	−0.16
inositol	−0.03	−0.59	0.01	0.28	−0.51
glucose	−0.38	0.63	0.00	−0.09	0.10
genistein	−0.38	−0.87	−0.31	0.39	−0.72
daidzein	−0.51	−0.88	−0.38	0.20	−0.55
stearic acid	0.37	0.43	−0.22	−0.71	0.42
melinamide	0.14	−0.77	−0.33	0.15	−0.23
ammonia	0.46	0.83	0.28	−0.48	0.63
